# Barriers and Facilitators to Vadadustat Use for Managing Anemia in Adult Patients Undergoing Dialysis: A Qualitative Evidence Synthesis

**DOI:** 10.7759/cureus.101071

**Published:** 2026-01-08

**Authors:** Piercarlo Minoretti, Simone Lista, Kayvan Khoramipour, Alejandro Santos-Lozano, Enzo Emanuele

**Affiliations:** 1 Occupational Health, Studio Minoretti, Oggiono, ITA; 2 Neurology, Miguel de Cervantes European University, Valladolid, ESP; 3 Health Sciences, Miguel de Cervantes European University, Valladolid, ESP; 4 Epidemiology and Public Health, Miguel de Cervantes European University, Valladolid, ESP; 5 Clinical Pathology, 2E Science, Robbio, ITA

**Keywords:** barriers, dialysis-related anemia, erythropoiesis-stimulating agents, facilitators, hypoxia-inducible factor, qualitative evidence synthesis, real-world, vadadustat

## Abstract

Anemia in patients on maintenance dialysis is routinely managed with erythropoiesis-stimulating agents (ESAs) and iron supplementation. Vadadustat, an oral hypoxia-inducible factor prolyl hydroxylase inhibitor (HIF-PHI) that stimulates endogenous erythropoietin and improves iron metabolism, presents a novel therapeutic alternative. Following its March 2024 U.S. Food and Drug Administration approval for adult patients on dialysis for at least three months, the clinical adoption of vadadustat continues to face significant implementation hurdles despite demonstrating non-inferiority to ESAs. In this study, we sought to identify and characterize the barriers and facilitators influencing the real-world integration of vadadustat for managing symptomatic anemia in adults undergoing dialysis for chronic kidney disease. We conducted a qualitative evidence synthesis, analyzing data from clinical trials, regulatory documents, and the nephrology literature. Our analysis identified 59 distinct barriers and 51 facilitators across healthcare systems, clinical practice, patient, and pharmacoeconomic domains. Prominent barriers include cardiovascular safety concerns, cost and reimbursement challenges, established ESA infrastructure, and prescriber unfamiliarity with the HIF-PHI drug class. Conversely, key facilitators comprise the convenience of oral administration, improved iron utilization, potential to overcome ESA hyporesponsiveness, and individual patient preference for oral therapy. Based on these findings, we propose a synthesis-derived multidisciplinary implementation framework built on six core principles: evidence-based patient selection, risk stratification and monitoring, shared decision-making, gradual transition strategies, interdisciplinary team engagement, and outcomes-based evaluation. Understanding these implementation determinants is crucial for optimizing anemia management in the dialysis population, ensuring that appropriate patient subgroups can benefit from vadadustat administration while balancing safety and cost-effectiveness.

## Introduction and background

Anemia is one of the most common and clinically significant complications of chronic kidney disease (CKD), affecting the vast majority of patients receiving maintenance dialysis [1]. Its pathogenesis is multifactorial, driven by insufficient erythropoietin (EPO) production, eryptosis, chronic inflammation, and both absolute and functional iron deficiency [2,3]. When inadequately treated, anemia contributes to significant morbidity, including fatigue, reduced exercise capacity, impaired cognitive function, and increased cardiovascular strain, ultimately diminishing quality of life [2]. For over three decades, the standard of care has centered on erythropoiesis-stimulating agents (ESAs), comprising recombinant human erythropoietin (epoetin alfa and beta) and longer-acting agents such as darbepoetin alfa [4]. While ESAs have transformed nephrology by markedly reducing transfusion requirements, their utility is limited by safety concerns at higher or supraphysiologic dosing [5], hyporesponsiveness in approximately 20% of patients [6], and substantial healthcare costs [7].

Vadadustat (Vafseo; Akebia Therapeutics, Inc., Cambridge, MA, USA) represents a mechanistically distinct approach to anemia management in CKD [8]. As a hypoxia-inducible factor prolyl hydroxylase inhibitor (HIF-PHI), vadadustat stabilizes HIF-1α and HIF-2α, activating a coordinated transcriptional program that promotes erythropoiesis [8,9]. This mechanism stimulates endogenous EPO production, increases intestinal iron absorption, and suppresses hepcidin to mobilize iron stores [10]. The Phase 3 INNO_2_VATE trial demonstrated that oral vadadustat is noninferior to darbepoetin alfa with respect to cardiovascular safety and correction and maintenance of hemoglobin concentrations in adult patients undergoing dialysis [11]. Based on these findings, vadadustat received regulatory approval for anemia in adult patients undergoing dialysis in multiple jurisdictions, including Japan, Australia, Korea, Taiwan, and the European Union [12]. The U.S. provided the most recent major approval on March 27, 2024 [8]. Despite regulatory clearance, real-world vadadustat adoption remains fragmented. The transition from established parenteral ESA regimens to oral HIF-PHI therapy poses multifaceted implementation challenges across clinical, operational, and economic domains, including workflow reconfiguration, modified monitoring protocols, safety surveillance adaptation, reimbursement uncertainty, limited prescriber familiarity with the drug class, and heterogeneous regulatory landscapes across jurisdictions [1,12-15]. Understanding what drives or impedes adoption is therefore essential for optimizing anemia management in appropriate patient subgroups while balancing safety and cost-effectiveness. This qualitative evidence synthesis addresses this implementation gap through three objectives. First, we systematically identify barriers currently limiting vadadustat adoption in adult patients undergoing dialysis. Second, we characterize facilitators that may support its integration across diverse dialysis settings. Third, we propose a synthesis-derived implementation framework to guide evidence-based patient selection and anemia care transition from ESAs to vadadustat.

## Review

Methods

Search Strategy and Data Sources

A literature search was conducted using PubMed, Embase, Web of Science, and Google Scholar databases to identify relevant publications from January 2015 through April 2025. The search strategy incorporated terms related to: (i) vadadustat and HIF-PHIs; (ii) ESAs; (iii) dialysis-dependent anemia; (iv) implementation, barriers, facilitators, and adoption; and (v) clinical practice, prescribing patterns, and healthcare delivery. Additional sources included regulatory documents from the U.S. Food and Drug Administration, European Medicines Agency, and Pharmaceuticals and Medical Devices Agency (Japan); nephrology society guidelines; health technology assessment reports; and conference proceedings from nephrology and hematology meetings. Eligible investigations included randomized controlled trials comparing vadadustat to ESAs; observational studies of vadadustat use in clinical practice; regulatory review documents; clinical practice guidelines; pharmacoeconomic analyses; and qualitative studies examining stakeholder perspectives on anemia management. Publications focusing exclusively on non-dialysis patients with CKD, other HIF-PHI agents without comparative reference to vadadustat, or basic science mechanistic studies without clinical implications were excluded. Notably, while we excluded purely mechanistic laboratory investigations (e.g., cell culture or animal studies), we retained mechanistic information from clinical sources when this knowledge directly informed understanding of implementation barriers or facilitators (e.g., hepcidin suppression explaining efficacy in ESA-resistant patients, or HIF pathway activation informing monitoring protocols).

Analytical Approach

A qualitative evidence synthesis approach was employed to identify and organize barriers and facilitators [16,17]. Relevant content was extracted from included sources and inductively coded to identify recurring themes. Codes were iteratively refined through constant comparison until thematic saturation was achieved. The resulting barriers and facilitators were organized using a multi-level taxonomy distinguishing between: (i) healthcare system factors, (ii) clinical practice factors, (iii) patient-level factors, and (iv) pharmacoeconomic factors. This classification recognizes that implementation determinants operate across different domains and often demonstrate bidirectional influences.

Results

Overview

Our qualitative synthesis identified 59 barriers (Table 1) and 51 facilitators (Table 2) influencing the adoption of vadadustat for managing anemia in adult patients undergoing dialysis. Notably, implementation determinants often exhibited context-dependent duality, functioning as barriers in one healthcare setting while serving as facilitators in another.

**Table 1:** Barriers to vadadustat adoption for managing anemia in adult patients undergoing dialysis ESA, erythropoiesis-stimulating agent; HIF-PHI, hypoxia-inducible factor prolyl hydroxylase inhibitor; IV, intravenous; KDIGO, Kidney Disease: Improving Global Outcomes.

Domain	Category	Specific Barriers
Healthcare System	Regulatory environment	Heterogeneous global regulatory landscape creating implementation complexity; Cardiovascular safety concerns raised in regulatory reviews; Boxed warnings for thrombotic events
	Reimbursement structure	Entrenched ESA reimbursement systems and payment bundling; Uncertain coverage policies for oral HIF-PHIs; Lack of distinct payment mechanisms for oral anemia therapies; Administrative burden of prior authorization requirements; Increased patient out-of-pocket costs in certain payer models
	Healthcare infrastructure	Facility infrastructure optimized for parenteral ESA administration; Entrenched inventory systems for ESAs and IV iron; Absence of pharmacy infrastructure for oral dispensing within dialysis units; Poor integration of oral medication tracking in dialysis electronic health records; Care fragmentation between dialysis centers and community pharmacies
Clinical Practice	Evidence gaps	Paucity of safety data beyond 2–3-year clinical trial horizons; Cardiovascular safety signals in pooled trial analyses; Ambiguity regarding optimal patient selection criteria; Insufficient data in key subpopulations (peritoneal dialysis, pediatrics); Lack of evidence guiding ESA-to-HIF-PHI switching protocols
	Prescriber factors	Clinician unfamiliarity with the HIF-PHI mechanism of action; Inertia of established ESA protocols developed over decades; Uncertainty managing novel adverse event profiles; Lack of confidence in dose titration and conversion ratios; Time constraints limiting uptake of new therapeutic education; Risk aversion regarding potential cardiovascular safety signals
	Monitoring and safety	Requirement for modified monitoring protocols versus standard ESA therapy; Potential drug-drug interactions; Ambiguity in managing elevated liver enzymes; Uncertainty regarding thrombosis surveillance protocols; Absence of standardized cardiovascular risk stratification tools
	Clinical guidelines	Absence of HIF-PHIs from KDIGO guidelines until the 2025 update; Limited endorsement in national nephrology society recommendations; Lack of shared consensus on indications for preferential use; Insufficient guidance on contraindications and risk stratification; No standardized switching algorithms from ESAs to vadadustat
Patient Level	Administration challenges	Adherence burden of oral medications in complex polypharmacy regimens; Coordination of dosing with dialysis schedules; Risk of missed doses due to high pill burden; Dysphagia or gastrointestinal intolerance; Complexity of medication reconciliation across transitions of care
	Knowledge and preferences	Low patient awareness of HIF-PHIs as therapeutic alternatives; Reluctance to adopt novel medications with limited track records; Preference for familiar ESA therapy established over the years; Misconceptions regarding efficacy of oral vs. injectable therapy; Health literacy and language barriers impeding education
	Clinical characteristics	Baseline cardiovascular comorbidities increasing safety concerns; History of venous thromboembolism; Hepatic impairment; Active malignancy or proliferative retinopathy; Gastrointestinal disorders compromising oral absorption
Pharmacoeconomic	Cost considerations	Higher acquisition costs vs. biosimilar ESAs in specific markets; Uncertainty regarding cost-effectiveness across diverse healthcare systems; Lack of real-world health economic models; Budget impact concerns for dialysis organizations; Pharmaceutical pricing negotiations and market volatility
	Resource allocation	Opportunity costs of implementing new management protocols; Resource requirements for staff training and education; Investment needs for medication reconciliation systems; Administrative overhead for prior authorization processing; Increased documentation and monitoring burden

**Table 2:** Facilitators for vadadustat adoption in managing anemia in adult patients undergoing dialysis ESA, erythropoiesis-stimulating agent; HIF, hypoxia-inducible factor; HIF-PHI, hypoxia-inducible factor prolyl hydroxylase inhibitor; IV, intravenous.

Domain	Category	Specific Facilitators
Healthcare System	Regulatory support	Regulatory approval in major markets; Accumulating post-marketing safety surveillance data; Precedent established for the HIF-PHI therapeutic class
	Policy initiatives	Recognition of need for ESA alternatives in guideline development; Quality improvement initiatives targeting ESA hyporesponsiveness; Shift toward patient-centered care models emphasizing choice
	Healthcare delivery	Alignment with transitions of care initiatives; Promotion of patient autonomy via self-administration
Clinical Practice	Clinical evidence	Demonstrated non-inferiority to ESAs in Phase 3 trials (INNO2VATE); Potential utility in ESA-hyporesponsive populations; Enhanced iron utilization reducing IV iron dependency; Improved hemoglobin stability with reduced variability; Suppression of hepcidin facilitating iron mobilization
	Mechanism of action	Physiologic erythropoiesis via coordinated HIF pathway activation; Stimulation of endogenous erythropoietin production; Enhanced intestinal iron absorption; Mobilization of sequestered iron stores; Potential anti-inflammatory effects of HIF activation; Pleiotropic effects on multiple HIF target genes
	Practical advantages	Oral administration eliminates injection burden; Flexible dosing schedules; Facilitates home administration independent of clinic visits; Elimination of cold chain storage requirements; Avoidance of injection site pain and reactions; Simplified administration for peritoneal dialysis patients
	Patient selection	Clear utility in ESA-hyporesponsive/resistant patients; Efficacy in functional iron deficiency; Alternative for patients with ESA intolerance/allergies; Option for patients with strong preference for oral therapy
Patient Level	Convenience factors	Elimination of painful injections; Increased therapy independence and control; Simplified regimen for home dialysis modalities; Dosing flexibility; Reduced dependence on in-center administration
	Quality of life	Empowerment through self-management; Alignment with patient preference for the oral route
	Clinical outcomes	Potential for improved hemoglobin stability; Reduced hemoglobin cycling vs. pulse ESA dosing; Lower transfusion requirements in select cohorts; Effective anemia correction in ESA-resistant cases; Potential reduction in inflammatory markers
Pharmacoeconomic	Cost-effectiveness potential	Reduced intravenous iron expenditure; Elimination of ESA wastage (unit-dose vs. multi-dose vials); Reduced nursing time for medication administration; Potential reduction in transfusion-related costs; Avoided costs of managing ESA hyporesponsiveness
	Healthcare efficiency	Elimination of cold chain logistics; Reduced sharps disposal volume; Lower occupational exposure risks (needlestick injuries)
	Market dynamics	Competitive pricing strategies for market entry; Price pressure from biosimilar competition

Regulatory and Reimbursement Factors

The evolving regulatory status of vadadustat has created a paradoxical landscape where policy decisions may act as both catalysts and constraints. Regulatory approvals across Japan, Australia, Korea, Taiwan, the European Union, and the U.S. [12] legitimize vadadustat as a therapeutic alternative to ESAs. However, significant regional variations in approval timing, labeling restrictions, and post-marketing surveillance mandates have resulted in fragmented uptake [9,18]. While some health systems are accumulating clinical experience [19], others, including the U.S., remain in a cautious exploratory phase. This tension is amplified by cardiovascular safety considerations. Although the INNO_2_VATE trial program demonstrated non-inferiority to darbepoetin alfa for both efficacy and cardiovascular safety in dialysis-dependent patients [11], the numerically higher, albeit statistically non-significant, incidence of vascular access thrombosis in this population [20], combined with unfavorable cardiovascular safety signals in non-dialysis patients [21], may still reinforce a conservative prescribing stance. In a clinical population with high baseline cardiovascular risk like patients on hemodialysis [22], even minor safety signals may prompt risk-averse prescribing, reinforcing the inertia of established ESA protocols. Reimbursement structures may further complicate this landscape. In contrast to ESAs, which are incorporated into the dialysis bundled payment under the ESRD Prospective Payment System [23], vadadustat, across several payer settings, is managed as a pharmacy benefit subject to prior authorization and standard prescription cost-sharing. Under Medicare, vadadustat currently receives separate reimbursement through the Transitional Drug Add-on Payment Adjustment (TDAPA) program [24], a temporary payment mechanism (typically two years from FDA approval) that provides additional reimbursement above the bundled rate for new drugs. Following TDAPA expiration (anticipated in 2026 for vadadustat), the drug must either transition into the bundled payment system or obtain permanent separate payment status, creating substantial reimbursement uncertainty that may inhibit long-term adoption planning by dialysis organizations. This "financing silo", which separates injectable facility-based therapies from pharmacy-dispensed oral agents, can misalign incentives among payers, providers, and patients. Consequently, such administrative and benefit-design complexity may blunt adoption even where clinical evidence supports use.

Clinical Evidence Gaps and Prescriber Inertia

The clinical evidence for vadadustat informs a complex risk-benefit calculation. While the INNO_2_VATE trial program established robust short-term efficacy of vadadustat for maintaining hemoglobin in dialysis-dependent patients [11], ESA hyporesponsiveness [25] and functional iron deficiency [26] remain historically challenging phenotypes. While emerging data with HIF-PHIs suggest potential advantages in these settings [27,28], ESA-resistant patients were largely excluded from INNO_2_VATE [11]. This creates a substantial evidence gap, i.e., the population most likely to benefit mechanistically from vadadustat's hepcidin-suppressing effects has the least robust trial evidence supporting its use. Consequently, any recommendations for vadadustat in ESA-hyporesponsive patients represent mechanistically informed extrapolation rather than evidence-based practice, requiring careful patient selection and enhanced monitoring. Another critical issue is the absence of long-term safety data beyond 2-3 years, specifically regarding thromboembolic events and the theoretical oncogenic risks of sustained HIF pathway activation [29]. Furthermore, pivotal dialysis trials predominantly enrolled patients with current or prior ESA exposure [11], resulting in relatively limited data on truly de novo vadadustat initiation in ESA-naive dialysis populations. For nephrologists, more than three decades of experience with ESAs have entrenched ESA-based dosing and titration practices [30], potentially contributing to clinical inertia and stable practice patterns around their use. Moreover, vadadustat’s distinct mechanism of action may introduce a new constellation of drug interactions and adverse events [31,32] that clinicians must learn to recognize and manage. In time-pressured clinical settings, the cognitive investment required to integrate novel pharmacological knowledge into practice workflows represents a non-trivial barrier. When weighed against the familiarity and established protocols surrounding ESAs, this learning requirement may tacitly favor therapeutic inertia [33], positioning vadadustat mainly as a specialized alternative rather than displacing ESAs as standard of care.

Patient-Level Considerations: Convenience Versus Adherence

Vadadustat presents a trade-off between convenience and the burden of adherence. On the one hand, the elimination of injections is a powerful facilitator, potentially addressing patient-reported outcomes regarding injection pain and anxiety [9,18]. The oral route also enhances autonomy, particularly for home dialysis patients [34]. On the other hand, this convenience relies on adherence, a known challenge in dialysis patients managing complex polypharmacy [35,36]. The addition of another oral agent introduces the risk of missed doses [37] and subsequent hemoglobin instability. While a thrice-weekly dosing schedule may mitigate pill burden, patient preference in real-world settings remains heterogeneous. Accordingly, some patients may prefer the “certainty” of provider-administered ESA injections during dialysis over self-managed complex oral therapy.

The Iron Metabolism Advantage and ESA Hyporesponsiveness

A key facilitator identified in our study is vadadustat’s distinct impact on iron metabolism [38]. Through HIF stabilization, the drug suppresses hepcidin and enhances iron mobilization from stores, potentially reducing dependence on intravenous iron supplementation [39]. This mechanism offers particular value for patients with ESA hyporesponsiveness (up to 20% of the dialysis population), a condition driven by inflammation-mediated iron sequestration and functional iron deficiency [40]. For this high-cost, high-risk subgroup where conventional ESA therapy proves inadequate, vadadustat may represent a mechanistically targeted therapeutic alternative [41]. This indication may serve as an implementation "beachhead", that is, a defined clinical niche where standard care inadequacy can justify the adoption of a novel agent, allowing providers to develop expertise before considering broader use.

Pharmacoeconomic Considerations

The economic value proposition of vadadustat remains incompletely characterized and context-dependent [42]. Notably, conventional acquisition cost analyses inadequately capture the drug’s full economic impact, as they fail to incorporate multiple offsetting factors across the care continuum [42]. Potential cost-saving mechanisms include reduced intravenous iron utilization, decreased nursing time for parenteral administration, elimination of ESA wastage from multidose vial discards, and reduced cold chain storage requirements. These facilitators, however, must be weighed against significant transactional barriers, including prior authorization administrative burden, fragmented reimbursement structures across payer types, and the financing asymmetry between facility-based and pharmacy-dispensed therapeutics [42]. Rigorous health economic modeling that incorporates these downstream cost effects, comprising opportunity costs associated with ESA-hyporesponsive patient management, is essential to establish vadadustat’s net economic value in heterogeneous real-world practice settings.

Implementation Framework for Vadadustat in Anemia Management Among Patients With Dialysis-Dependent Chronic Kidney Disease

**Figure 1:**
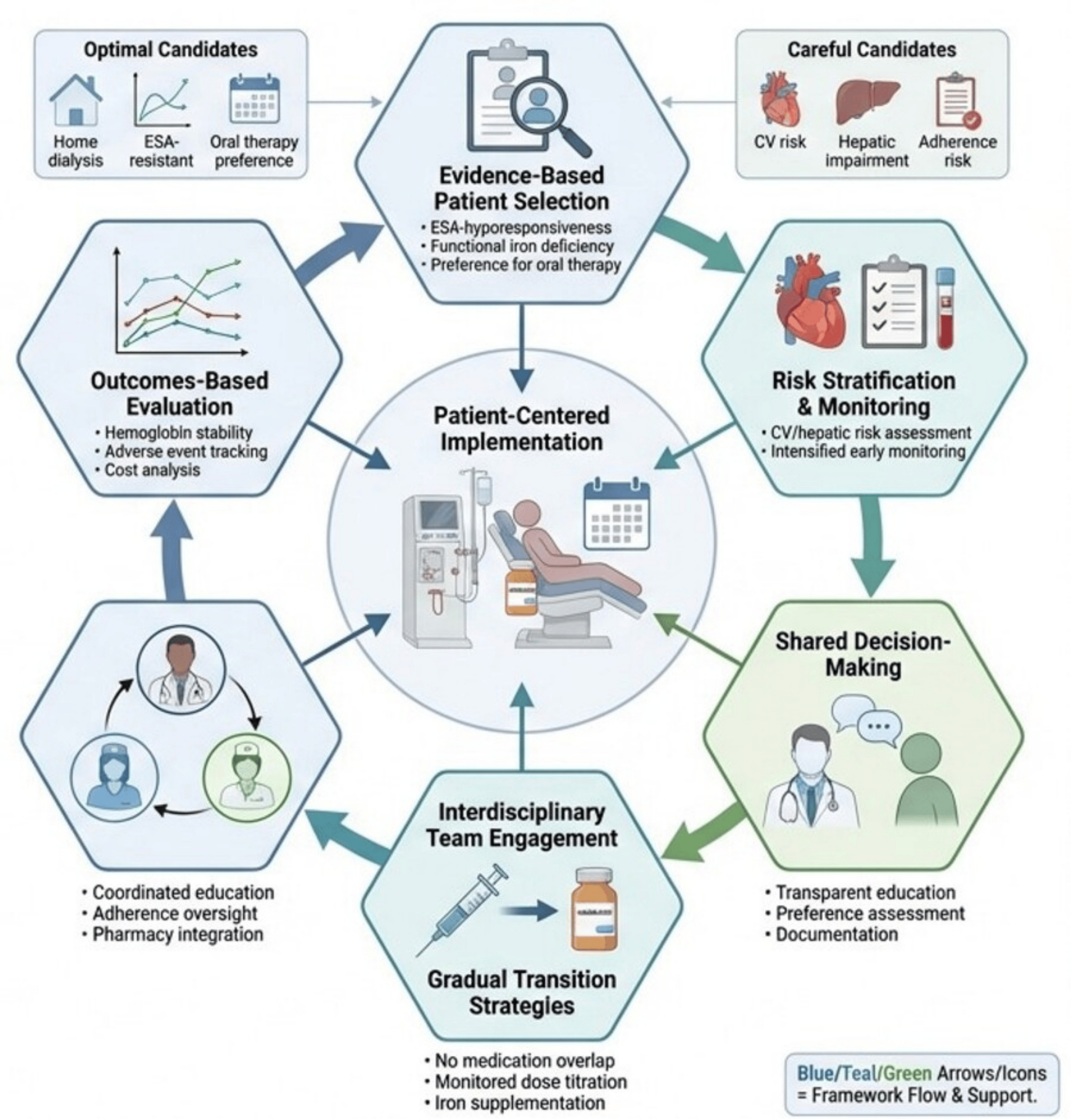
Synthesis-derived multidisciplinary framework for patient-centered implementation of vadadustat in adults receiving dialysis The proposed framework translates barriers and facilitators identified through qualitative evidence synthesis into operational implementation guidance; however, it has not been empirically tested or validated. In general, the framework integrates four sequential phases based on qualitative synthesis of real-world barriers and facilitators. Initial patient selection identifies optimal candidates (home dialysis, ESA hyporesponsiveness, preference for oral therapy) and those requiring cautious use (elevated cardiovascular risk, hepatic impairment, adherence concerns). Risk stratification and early monitoring of cardio-hepatic status and laboratory parameters inform iterative shared decision-making through transparent education, preference assessment, and documentation of treatment plans. Interdisciplinary team engagement supports gradual ESA-to-vadadustat transition via coordinated provider and nursing education, pharmacy integration, avoidance of overlapping ESA exposure, dose titration, and iron optimization. Outcomes-based evaluation assesses hemoglobin stability, adverse events, and costs, with findings feeding back to refine earlier phases. Arrows indicate directional flow; color schemes denote supporting components centered on the dialysis encounter. (*Created with FigureLabs.ai; Image Credit: Enzo Emanuele*). ESA, erythropoiesis-stimulating agent

Based on the identified barriers and facilitators, we propose a synthesis-derived multidisciplinary framework for implementing vadadustat in appropriate patient populations (Figure 1). This framework translates our qualitative evidence synthesis into operational guidance by systematically mapping implementation determinants to actionable principles. Notably, this framework has not been empirically validated and represents expert interpretation of available evidence rather than tested implementation science. In general, the framework is predicated on six core principles that integrate clinical evidence, risk mitigation strategies, and stakeholder engagement to optimize therapeutic outcomes while minimizing potential harms. Future research should prospectively evaluate this framework's feasibility, fidelity, and effectiveness in diverse dialysis settings.

Principle 1: Evidence-Based Patient Selection

Not all dialysis patients can be considered suitable candidates for vadadustat therapy. At present, use should be targeted to individuals most likely to derive clinical benefit while limiting exposure among those at higher risk of adverse events. Potentially appropriate candidates include patients wishing to avoid injections (e.g., because of needle phobia, limited nursing support, or home dialysis) and those receiving peritoneal dialysis [34]. Patients with functional iron deficiency or inflammation‑related ESA hyporesponsiveness may represent candidates based on mechanistic rationale and emerging post-marketing data [27,28,41], though it must be acknowledged that this population was systematically excluded from pivotal trials [11]. As vadadustat improves iron utilization parameters (lower hepcidin and ferritin, higher total iron binding capacity) while raising hemoglobin [43], the hepcidin-suppressing mechanism provides biological plausibility for efficacy in this setting. However, this represents hypothesis-generating extrapolation rather than Level 1 evidence. Use in ESA-hyporesponsive patients therefore requires explicit acknowledgment of the limited evidence base during shared decision-making, enhanced monitoring protocols, and ideally enrollment in registry studies or pragmatic trials to generate real-world safety and efficacy data. Additional suitable candidates include those with documented allergic reactions to ESAs [44]. Conversely, several subgroups warrant caution and may be considered suboptimal candidates for vadadustat initiation. Patients with recent cardiovascular events, including myocardial infarction, stroke, or venous thromboembolism, require careful evaluation given unfavorable safety signals in non‑dialysis populations [21] and possible increases in thromboembolic events in dialysis cohorts [11]. Those with active malignancies or proliferative retinopathy also warrant scrutiny because of theoretical concerns related to hypoxia-inducible factor activation in neoplastic or angiogenic processes [45]. Finally, patients with documented poor adherence to oral therapies may face reduced efficacy and heightened safety risks.

Principle 2: Monitoring Protocols

Transitioning patients to vadadustat from conventional ESAs requires the adoption of a modified surveillance framework. This protocol entails hemoglobin monitoring every two to four weeks during the initiation and dose-titration phase, followed by monthly assessments upon achieving therapeutic stability [46]. A key differentiator from ESA therapy is the hepatic safety monitoring, which may involve monthly liver enzyme assessments for the first three months and subsequent quarterly evaluations [47]. To ensure optimal hematopoietic response, iron stores must be reassessed quarterly [48]. Finally, this framework should be expanded beyond traditional protocols to include continuous cardiovascular symptom surveillance, due to the drug’s safety profile, and systematic verification of medication adherence, reflecting its oral route of administration [8].

Principle 3: Shared Decision-Making

Implementation frameworks must position patients as active partners in therapeutic decision-making rather than passive recipients of prescriber choices [49]. This requires provision of balanced, evidence-based information comparing vadadustat with conventional ESA therapy, including transparent discussion of clinical trial data, comprising both efficacy outcomes and safety considerations. In this scenario, clinicians should systematically explore patient preferences regarding oral versus injectable medication administration, recognizing that individual values and priorities may diverge from clinical trial-level evidence. In addition, the novelty of vadadustat relative to established ESA therapy may generate patient concerns that require empathetic acknowledgment and evidence-based responses [42]. Assessment of patient ability and willingness to adhere to oral medication regimens represents a key prerequisite for successful implementation, as therapeutic failure due to non-adherence may expose patients to risks without corresponding benefits. Cultural and linguistic factors must also be considered in educational interventions to ensure genuine informed consent rather than perfunctory documentation. The entire shared decision-making process, including patient comprehension and agreement, warrants formal documentation in the medical record.

Principle 4: Optimal Transition Strategies

For patients transitioning from ESAs to vadadustat, a direct conversion protocol without medication overlap is recommended [9]. The rationale for avoiding overlap derives from the potential for additive erythropoietic effects when combining exogenous ESAs with endogenous erythropoietin stimulation via HIF pathway activation, which may precipitate rapid hemoglobin rises and associated thrombotic risks [9]. Vadadustat should therefore be initiated at the time the next scheduled ESA administration would have been due. Current evidence supports a standard starting dose of 300 mg once daily [29]. However, this uniform approach does not account for substantial variation in prior ESA requirements; in this context, a patient receiving 2,000 units epoetin alfa weekly likely has different erythropoietic needs than one requiring 40,000 units weekly. Unfortunately, evidence-based ESA-dose-dependent conversion algorithms do not currently exist, necessitating individualized clinical judgment. Patients at extremes of ESA dosing warrant particular attention: those on very high doses (suggesting ESA hyporesponsiveness) may require closer hemoglobin monitoring to detect either inadequate response necessitating more rapid up-titration, or conversely, unexpected sensitivity requiring dose reduction. Hemoglobin monitoring should be intensified during the first four to six weeks following conversion to identify suboptimal response or excessive hemoglobin velocity [39]. Dose adjustments are implemented in 150-mg increments (available doses: 150 mg, 300 mg, 450 mg, maximum 600 mg daily) based on hemoglobin trajectory, with four-week titration intervals generally recommended to allow full pharmacodynamic effects to manifest before further modification [34]. Iron supplementation must be maintained throughout the transition and beyond, with regular assessment of ferritin and transferrin saturation to ensure adequate iron availability for erythropoiesis, particularly important given vadadustat's hepcidin-suppressing effects that enhance iron utilization [11]. The transition from ESAs to vadadustat represents a fundamental shift in anemia management paradigm. Unlike ESAs, which are administered one to three times weekly via parenteral injection, vadadustat requires once-daily oral administration [42]. This shift carries dual implications: it offers potential convenience for patients preferring oral therapy and enhanced autonomy for home dialysis patients, while simultaneously creating adherence challenges for those unaccustomed to daily medication regimens. Missed doses may result in hemoglobin fluctuations and suboptimal anemia control, underscoring the importance of adherence assessment and patient education during the transition period.

Principle 5: Interdisciplinary Team Engagement

Successful vadadustat implementation requires coordinated engagement across the healthcare team, extending well beyond individual prescribing decisions. At the center of this collaborative approach, nephrologists retain primary responsibility for evidence-based patient selection, dose optimization, and adverse event management [42]. They also serve a crucial educational function, training staff on how HIF-PHI mechanisms fundamentally differ from ESA pharmacology. Building on this clinical foundation, dialysis nurses should operationalize therapy through direct patient education and adherence monitoring. They may also coordinate with pharmacy services to ensure uninterrupted medication availability, a critical link in the care continuum. Notably, pharmacists should complement this clinical oversight by providing essential expertise in medication reconciliation [50].

Principle 6: Outcomes-Based Evaluation

Successful vadadustat implementation demands systematic outcomes evaluation across multiple domains to assess real-world effectiveness, safety, patient experience, and economic impact [29]. This comprehensive assessment should begin with clinical efficacy metrics, tracking hemoglobin target achievement relative to pre-vadadustat baselines, hemoglobin stability measured by reduced cycling between supra- and sub-target values, and ESA-to-vadadustat conversion success rates. More fundamentally, changes in intravenous iron utilization, reflecting the drug’s hepcidin suppression mechanism, and transfusion requirements serve as ultimate measures of anemia control [11], demonstrating whether the novel mechanism translates to meaningful real-world clinical benefits. In parallel, safety monitoring should determine the risk-benefit balance in routine dialysis settings through systematic capture of adverse events, specifically major adverse cardiovascular events, thromboembolic complications, and hepatotoxicity signals [51]. Beyond discrete adverse events, discontinuation rates provide crucial insight into tolerability outside controlled trial environments, while hospitalization rates can offer a broader measure of clinical stability that captures both efficacy and safety dimensions. Complementing these objective clinical measures, patient-reported outcomes represent an essential but commonly neglected evaluation domain [52]. Treatment satisfaction assessments and quality of life measures may provide the patient perspective necessary for truly patient-centered care, while validated adherence measurements and assessments of perceived convenience comparing oral versus injectable therapy would offer significant insights into the feasibility and sustainability of long-term implementation. Finally, economic metrics complete the evaluation framework by translating clinical and patient outcomes into resource utilization terms [42]. While acquisition costs represent the most visible factor, comprehensive assessment must account for total anemia management costs, including reductions in intravenous iron expenditure and nursing time previously devoted to ESA administration. Healthcare utilization patterns, comprising emergency department visits attributable to anemia complications, may capture downstream economic consequences that extend beyond pharmaceutical spending, while formal cost-effectiveness analyses comparing vadadustat to ESAs in specific subpopulations prove essential for informing formulary positioning and resource allocation decisions that ensure clinical benefits align with healthcare system sustainability [42].

Discussion

In this qualitative evidence synthesis, we identified numerous barriers and facilitators to vadadustat adoption in dialysis-associated anemia across healthcare systems, clinical practice, patient, and pharmacoeconomic domains, with substantial interdependencies among factors. Among the diverse implementation challenges identified, persistent uncertainty regarding cardiovascular safety emerges as perhaps the most significant barrier to widespread vadadustat implementation. Although pivotal trials in dialysis-dependent patients (INNO_2_VATE) demonstrated non-inferiority for major adverse cardiovascular events [11], safety signals observed in non-dialysis populations (PRO_2_TECT) [21] and numerically higher vascular access thrombosis events in dialysis patients [20] have created sufficient concern to engender real-world prescriber hesitancy, even in countries where the agent is available. Notably, the cardiovascular controversy highlights a broader challenge in nephrology therapeutics. Dialysis patients represent a high-risk population with high cardiovascular event rates regardless of specific interventions [53]. In this scenario, separating drug-related cardiovascular risk from background population risk demands large, well-powered, long-term studies, which remain expensive, time-consuming, and operationally complex. These evidence gaps may create clinical uncertainty, prompting clinicians and patients to default to established ESAs despite their known limitations. Nonetheless, rather than debating universal adoption or avoidance of vadadustat, adherence to U.S. FDA-mandated labeling and risk stratification approaches must identify patients most and least likely to benefit, tailoring therapy to individual cardiovascular risk profiles. Implementing such strategies, however, requires granular patient-level data and clinical decision support tools that are continuously evolving following the drug's 2024 U.S. approval. These safety considerations, codified by the FDA's Boxed Warning regarding increased risk of death, myocardial infarction, stroke, and venous thromboembolism [54], currently point toward a more targeted implementation strategy rather than positioning vadadustat as a wholesale replacement for ESAs in all dialysis patients. 

ESA-hyporesponsive patients represent a compelling yet problematic use case for vadadustat [42]. This population demonstrates limited response to current therapy, often requires very high ESA doses (with attendant costs and potential risks), and still frequently fails to achieve hemoglobin targets despite aggressive treatment. Mechanistically, vadadustat's hepcidin suppression directly addresses the inflammation-mediated iron sequestration that underlies ESA resistance [27], providing strong biological rationale for efficacy. However, a critical evidence gap undermines this rationale, i.e., ESA-resistant patients were systematically excluded from pivotal trials [11], creating a paradox whereby the subgroup most likely to benefit mechanistically has the weakest empirical evidence base. Our framework's inclusion of ESA-hyporesponsive patients therefore represents mechanistically informed extrapolation rather than evidence-based practice. Clinicians considering vadadustat in this population should explicitly acknowledge this evidence limitation during shared decision-making, implement intensified monitoring protocols, and ideally contribute to registry-based outcomes research to address this knowledge gap. Beyond ESA-resistant patients, individuals with strong preferences for oral therapy or those on home dialysis modalities [34] may represent more straightforward implementation niches. In these populations, vadadustat's facilitators (i.e., oral administration, dosing flexibility, elimination of injection burden, and improved iron utilization) offer clear practical advantages with less reliance on extrapolated efficacy data. Beginning with these focused applications allows accumulation of real-world experience, refinement of monitoring protocols, and development of prescriber comfort before considering broader implementation.

Beyond clinical evidence considerations, our analysis revealed that healthcare system factors, including current regulatory environment, reimbursement structure, and delivery infrastructure, represent major barriers. The current system for anemia management in dialysis has been optimized over decades for ESA delivery, including medication procurement and inventory management systems, nursing workflows, cold chain storage, administration documentation, and payment models all reflect the established paradigm [55]. Introducing oral vadadustat disrupts these established processes, requiring major investments in new workflows, medication dispensing systems, and reimbursement arrangements. For dialysis organizations operating on thin margins, the transaction costs of changing anemia management approaches, even if the new approach may potentially offer economic advantages in the long-term, may exceed organizations’ capacity or willingness to implement change. This healthcare system inertia is particularly powerful in the U.S., where long-term bundled payment uncertainty (post-TDAPA period), specifically, the unknown reimbursement status after the two-year TDAPA period expires, and separate ESA reimbursement may create financial structures that might not readily accommodate oral anemia medications indefinitely. In contrast, healthcare systems with different payment models (such as global budgets or capitated arrangements) may find it easier to adopt vadadustat if the total cost of anemia management (medication plus monitoring plus adverse event management) proves favorable compared to ESAs.

Navigating these system-level barriers while addressing clinical uncertainties requires placing patients at the center of implementation decisions [56]. The patient-level barriers and facilitators identified in our synthesis underscore the importance of shared decision-making in anemia management. Patient preferences regarding oral versus injectable medications can vary considerably, influenced by individual experiences, cultural factors, health literacy, and trust in different treatment modalities. While some patients perceive substantial benefit from eliminating injections and gaining autonomy through oral self-administration, others may prefer the certainty and routine of receiving ESAs during dialysis. In this scenario, effective implementation of vadadustat requires frank discussion of uncertainties, particularly regarding long-term cardiovascular safety, and exploration of what matters most to individual patients. For some, the potential convenience benefits and reduced injection burden can justify accepting some uncertainty about long-term cardiovascular outcomes (particularly if their cardiovascular risk profile is relatively favorable). For others, the proven track record of ESAs can easily outweigh any potential advantages of oral therapy. This patient-centered approach is in accordance with broader movements toward shared decision-making in nephrology [57], recognizing that evidence-based medicine must be integrated with patient values and preferences rather than applied uniformly regardless of individual circumstances. Given the complexity of vadadustat’s risk-benefit profile, decision aids and structured communication tools become essential for translating clinical evidence into patient-centered discussions about treatment selection.

Clinical and policy deliberations will also depend on real-world evidence addressing the substantial gap between trial efficacy and routine practice effectiveness. Post-marketing data on safety, adherence, resource utilization, and patient outcomes, particularly cardiovascular events in unselected populations, efficacy in ESA-resistant patients, and economic impact following TDAPA expiration, will inform implementation strategies and patient selection algorithms. While registry studies, pragmatic trials, and administrative database analyses provide suitable methodologies, generating this evidence will require sustained collaboration among drug manufacturers, regulators, dialysis organizations, and researchers, all partnerships that remain difficult to establish and sustain.

This qualitative evidence synthesis has several limitations. First, the evidence base relies predominantly on clinical trial data and regulatory documents, with limited real-world experience given vadadustat's recent market entry. Second, our analysis reflects evidence available through December 2025; accumulating post-marketing data may substantially reshape implementation considerations in the future. Third, barriers and facilitators likely vary across healthcare systems, patient populations, and geographic contexts. Fourth, socioeconomic factors, including medication affordability, access to nephrology care, and health system capacity, can fundamentally influence implementation but vary considerably across settings; future research should examine how these factors moderate the barriers and facilitators identified here. Fifth, the implementation framework presented in Figure 1 represents synthesis-derived expert interpretation rather than empirically validated guidance. While the framework systematically translates identified barriers and facilitators into operational principles, it has not undergone prospective testing for feasibility, implementation fidelity, or effectiveness outcomes. Real-world pilot testing and iterative refinement are therefore essential before widespread adoption. Finally, interpretive bias in coding and thematic analysis represents an inherent limitation of qualitative synthesis methodology.

## Conclusions

Vadadustat adoption in dialysis-dependent anemia faces substantial barriers spanning regulatory uncertainty, clinical evidence gaps, healthcare system inertia, and pharmacoeconomic constraints. However, oral administration, potential efficacy in ESA-resistant patients, improved iron utilization, and specific patient preferences represent meaningful advantages. Rather than universal adoption or avoidance, optimal implementation targets specific populations where benefits may outweigh risks, including ESA-hyporesponsive patients (acknowledging limited direct trial evidence but strong mechanistic rationale), those with functional iron deficiency, individuals preferring oral therapy, and home dialysis patients. The synthesis-derived multidisciplinary framework presented here, emphasizing evidence-based patient selection, risk stratification, shared decision-making, and outcomes monitoring, offers a proposed pragmatic pathway for clinicians and systems navigating vadadustat adoption that requires prospective validation in diverse clinical settings. This approach will also require refinement as real-world evidence accumulates and regulatory landscapes evolve. Notably, the objective is not to advocate for or against vadadustat universally, but to ensure that patients and clinicians can access multiple therapeutic options, make informed decisions based on available evidence, and deliver personalized care that optimizes both clinical outcomes and patient experience.
